# HIV-1 molecular epidemiology and drug resistance-associated mutations among treatment-naïve blood donors in China

**DOI:** 10.1038/s41598-020-64463-w

**Published:** 2020-05-05

**Authors:** Junpeng Zhao, Xiaoting Lv, Le Chang, Huimin Ji, Barbara J. Harris, Lu Zhang, Xinyi Jiang, Fei Guo, John Hackett, Peng Yin, Gavin A. Cloherty, Mary A. Rodgers, Lunan Wang

**Affiliations:** 1 National Center for Clinical Laboratories, Beijing Hospital, National Center of Gerontology, Institute of Geriatric Medicine, Chinese Academy of Medical Sciences, Beijing, P. R. China; 2 Graduate School, Peking Union Medical College, Chinese Academy of Medical Sciences, Beijing, P. R. China; 3 Abbott Laboratories, Research and Development, Shanghai, P.R. China; 4 Abbott Laboratories, Infectious Disease Research, Abbott Park, IL USA

**Keywords:** Clinical microbiology, Retrovirus, Viral epidemiology

## Abstract

Surveillance of human immunodeficiency virus (HIV) molecular diversity and drug resistance-associated mutations (DRMs) among treatment-naïve blood donors is critical for monitoring viral evolution and blood safety. From 2016-2017, 199 plasma samples were collected from 24 blood centers and confirmed as HIV viral load positive or serologically reactive in National Centers for Clinical Laboratories (NCCL), of which 179 were sequenced and subtyped in the *gag*, *protease* (PR)-*reverse transcriptase* (RT), *integrase* (IN) and/or *envelope* (*env*) regions. DRMs in PR-RT and IN regions were analyzed in Stanford HIVdb Program. The majority of subtypes were circulating recombinant form (CRF) 07_BC (34.6%) and CRF01_AE (32.4%); many unique recombinant forms (URFs) (39, 21.8%) and other rare CRFs were observed in the study. Notably, CRF02_AG and CRF06_cpx strains typically found in Africa were firstly identified amongst Chinese blood donors. DRMs were common, with 28 of 179 (15.6%) specimens carrying DRMs, including the PR N88S and RT K103N mutations, which have been implicated in elevated resistance to antiretroviral drugs. Furthermore, 4 HIV-1 isolates (2.4%, 4/168) had surveillance drug-resistance mutation (SDRM), including 3 nonnucleoside*reverse transcriptase* inhibitors (NNRTI) SDRMs (1 K101E, 2 K103N) and 1 *protease* inhibitor (PI) SDRM (M46I). The HIV viral diversity among blood donors observed in this study suggest that ongoing HIV-1 recombination is becoming progressively complex in China, and lots of DRMs found in the study exacerbate the primary drug resistance landscape, which highlight the necessity of timely genotypic drug resistance monitoring and molecular surveillance of HIV-1 among blood donors.

## Introduction

According to a recent report from the National Center for Acquired Immunodeficiency Syndrome/Sexually Transmitted Disease (AIDS/STD) Control and Prevention (China CDC), there were approximately 849,602 people living with HIV and 262,442 reported HIV-associated deaths in China as of September, 2018^1^. Epidemiological evidence indicates that the HIV epidemic has shifted from high risk populations into general populations in China, including blood donors^2,3^. In 2003, the “Four Frees and One Care” policy was implemented to reduce AIDS-related mortality in China by providing free antiretroviral drugs, including highly active antiretroviral therapy^4^. However, the effectiveness of antiretroviral therapy (ART) may be limited by the transmission of HIV drug resistant strains to ART-naïve patients^5^, which is a major obstacle to viral suppression^6,7^. Therefore, the World Health Organization strongly recommends surveillance of transmitted drug resistance (TDR) amongst HIV infected populations^8^. Characterization of HIV genetic diversity and TDR among volunteer blood donors (treatment-naïve populations) is essential for monitoring viral evolution and optimal ART selection, both have important roles in blood safety^9^. Moreover, viral diversity has the potential to impact the sensitivity and accuracy of HIV blood screening tests, potentially putting the blood supply at risk as new strains emerge^10–12^. Molecular epidemiological analyses have been powerful tools to investigate the origin and evolution of HIV-1 variants around the world^13,14^. In China, CRF07_BC, CRF01_AE, CRF08_BC and subtype B are the four most prevalent HIV-1 strains, according to a nationwide molecular epidemiologic survey by China CDC between 2006–2008^15^. In 2012, the US National Heart, Lung and Blood Institute (NHLBI) initiated a surveillance study of the molecular epidemiology of HIV among Chinese blood donor population as an important part of the Recipient Epidemiology and Donor Evaluation Study^9^. Since this study focused on only 5 blood centers, limited HIV diversity data are currently available. Furthermore, the prevalence of TDR amongst Chinese blood donors is unknown.

In this study, HIV diversity and DRM prevalence were examined among Chinese blood donors from 24 blood screening laboratories between January 2016 and December 2017, covering 17 provinces or municipalities, including all geographic regions (North China, South China, Northwestern District of China and Qinghai-Tibet region). This large-scale study provides the most recent and comprehensive data on HIV-1 molecular epidemiology and TDR among Chinese blood donors, which may inform optimal delivery of ART, improve HIV screening strategies, and serve as a resource for blood centers in China.

## Results

### Demographic characteristics of blood donors

A total of 199 blood donors confirmed as HIV-1 seropositive or viral load positive were enrolled in this study, and 179 donations were successfully sequenced. From the 179 plasma samples, 168 *gag*, 170 IN, 168 PR-RT and 166 *env* sequences were generated by Sanger methods. Demographic information from all participants is summarized in Table 1. Notably, the majority of study participants were male (92.2%, 165/179), first-time donors (62.0%, 111/179), Han (95.0%, 170/179) and aged 18–35 years old (73.2%,131/179). Most of the HIV-1 infected blood donors had a lower educated level (Associate degree and secondary school or below: 80.4%, 144/179).

**Table 1:** Molecular epidemiological characteristics of HIV-1 infected blood donors.

Characteristics	Total N = 179
**Age (years old)**	
18–25	49 (27.4%)
26–35	82 (45.8%)
36–45	25 (14.0%)
46–55	20 (11.2%)
>55	3 (1.7%)
**Gender**	
Female	14 (7.8%)
Male	165 (92.2%)
**Previous donation history**	
Repeat donor	68 (38.0%)
First-time donor	111 (62.0%)
**Ethnicity**	
Minority	9 (5.0%)
Han	170 (95.0%)
**Education**	
Masters/Bachelor degree	35 (19.6%)
Associate degree	66 (36.9%)
Secondary school or below	78 (43.6%)
**Genotype**	
CRF07_BC	62 (34.6%)
CRF01_AE	58 (32.4%)
B	9 (5.0%)
CRF08_BC	3 (1.7%)
CRF02_AG	1 (0.6%)
CRF55_01B	2 (1.1%)
CRF59_01B	1 (0.6%)
CRF65_cpx	1 (0.6%)
CRF67_01B	1 (0.6%)
CRF79_0107	1 (0.6%)
CRF85_BC	1 (0.6%)
URF	39 (21.8%)

## HIV-1 subtype classification

**Figure 1:**
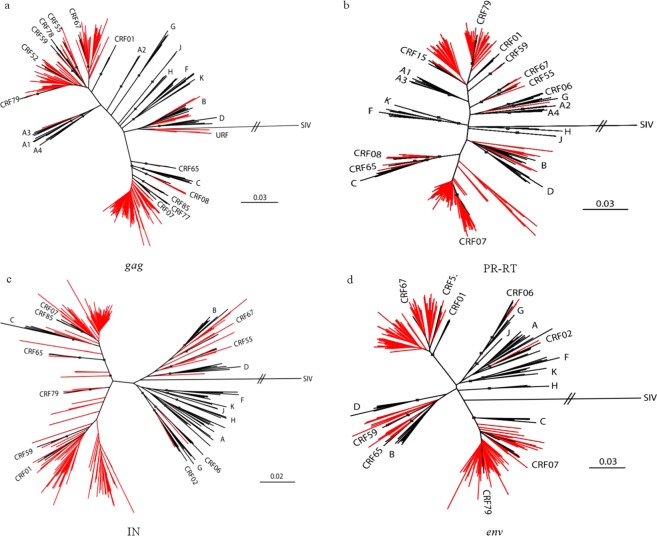
Neighbor-joining phylogenetic tree analysis of HIV-1 isolates in blood donors. Sequences from HIV-1 infected blood donors and references are respectively in red and black in the trees and boxes indicate relevant nodes with >70 bootstrap. (**a**) Phylogenetic tree analysis of *gag* sequences. (**b**) Phylogenetic tree analysis of PR-RT sequences. (**c**) Phylogenetic tree analysis of IN sequences. (**d**) Phylogenetic tree analysis of *env* sequences.

**Figure 2:**
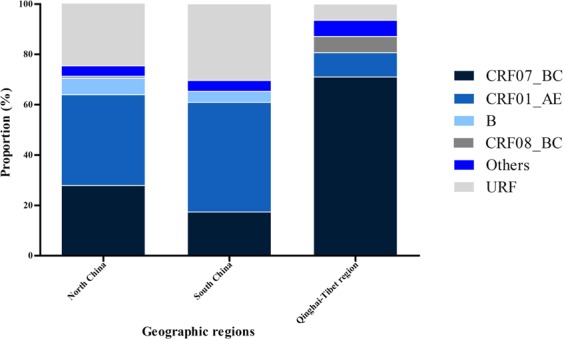
The geographic distribution of HIV- I subtype among blood donors. (**a**) The Northwestern District of China was excluded on account of the small sample sizes. Others represent the rare CRFs including CRF02_AG, CRF55_01B, CRF59_01B, CRF65_cpx, CRF67_01B, CRF79_0107 and CRF85_BC.

After initial HIV-1 genotyping by the HIV BLAST tool, REGA HIV-1 Subtyping Tool-Version 3.0 and jpHMM program, the specimen classifications were performed using phylogenetic inference of the *gag*, PR-RT, IN and *env* regions (Fig. 1). HIV-1 subtype was confirmed by the consistent results from the subtyping tools above, between different gene regions. All potential unique recombinant sequences (the sequences with inconsistent subtyping results from the tools above) were further analyzed by SimPlot 3.5.1 software to determine recombination breakpoints (Fig. S1) and subtypes. Recombinant composition of URFs were displayed in Table 2. It is noted that a limitation of the recombinant HIV-1 drawing tool used to generate Fig. S1 does not allow CRF labels other than CRF01_AE or CRF02_AG. Therefore, regions that were classified as a CRF with a strong bootstrap value and branching pattern are labeled as the parental strains for that CRF, including regions where no recombinant breakpoints are present. In addition to subtype B and C sequences, a diverse set of CRFs were identified amongst the sequenced regions, including CRF01_AE, CRF02_AG, CRF06_cpx, CRF07_BC, CRF08_BC, CRF15_01B, CRF52_01B, CRF55_01B, CRF59_01B, CRF65_cpx, CRF67_01B, CRF77_cpx, CRF78_cpx, CRF79_0107, CRF83_cpx, and CRF85_BC. The majority of specimens were classified as CRF07_BC (34.6%, 62/179) or CRF01_AE (32.4%, 58/179), with URFs being nearly as common as these CRFs (21.8%, 39/179). Although the relative prevalence of each classification varied between geographic regions in China (Number of samples ≥10), URFs were present in all locations (Fig. 2).

**Table 2:** Recombinant composition of URFs.

Sample ID	Provinces or municipalities	Region classifications (*gag*/PR-RT/IN/*env*)*
Changchun-001	Jilin	07/79/01/01
Changchun-018	Jilin	B/B/01-B/-
Changchun-066	Jilin	07/07/07/B
Chongqing-009	Chongqing	07/-/01–07/07
Chongqing-CWB	Chongqing	07/07/07/01
Harbin-010	Heilongjiang	07/79/01/07
Harbin-017	Heilongjiang	07/07/83/01
Harbin-022	Heilongjiang	-/55/-/01
Harbin-035	Heilongjiang	01/79/01/07
Harbin-045	Heilongjiang	07/07/83/01
Henan-005	Henan	67/07/01/01
Henan-006	Henan	-/79/07/07
Henan-007	Henan	07/01/07/07
Henan-011	Henan	67/79/01/01
Henan-012	Henan	67/01-07/01/07
Henan-013	Henan	B/-/B/01
Henan-014-5HN	Henan	01/01-07/01-07/79
Henan-019	Henan	55-B/55/55/55
Jiangsu-003	Jiangsu	67/67/01/67
Jiangsu-007	Jiangsu	67/15/79/01
Jiangsu-009	Jiangsu	07/B/07/07
Liaoning-005	Liaoning	52/-/B/01
Liaoning-011	Liaoning	78/79/01-07/01
Liaoning-012	Liaoning	01/79/01/07
Shaanxi-005-5SX	Shaanxi	-/07/-/01
Shaanxi-007	Shaanxi	-/79/79/01
Shaanxi-014-5SX	Shaanxi	07/01-07/79/01-B
Shaanxi-014	Shaanxi	01/A-C/01/01
Shaanxi-015	Shaanxi	A3/79/06/06
Shaanxi-017	Shaanxi	67/07/07/06
Shenzhen-006	Guangdong	-/07/01-07/-
Shenzhen-012	Guangdong	67/-/-/07
Shenzhen-012-2018	Guangdong	01/55/01/01
Tongzhou-025	Beijing	01/79/01/65
Tongzhou-035	Beijing	01/07/01/07
Tongzhou-115	Beijing	01/B/01/01
Tongzhou-126	Beijing	67/01/01/79
Wuhan-004	Hubei	07/07/07/01
Yuncheng-007	Shanxi	07/07/83/B

^*^CRFs were shown as simplified numbers.

-: no sequence.

**Figure 3:**
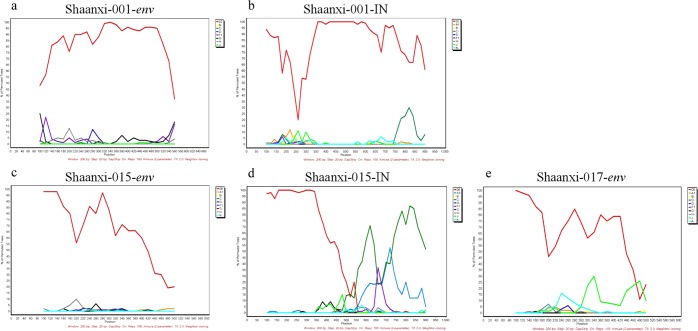
Bootscan plots of the three rare recombinant partial-genome sequences from the HIV-1 infected blood donors. Each bootscan plot was performed with Kimura-2 model of nucleotide substitution with a window size of 200 and a step size of 20. The color-coded key represents the different subtypes, sub-subtypes and CRFs of HIV-1. (**a**) Shaanxi-001 *env* sequence. (**b**) Shaanxi-001 IN sequence. (**c**) Shaanxi-015 *env* sequence. (**d**) Shaanxi-015 IN sequence. (**e**) Shaanxi-017 *env* sequence.

**Figure 4:**
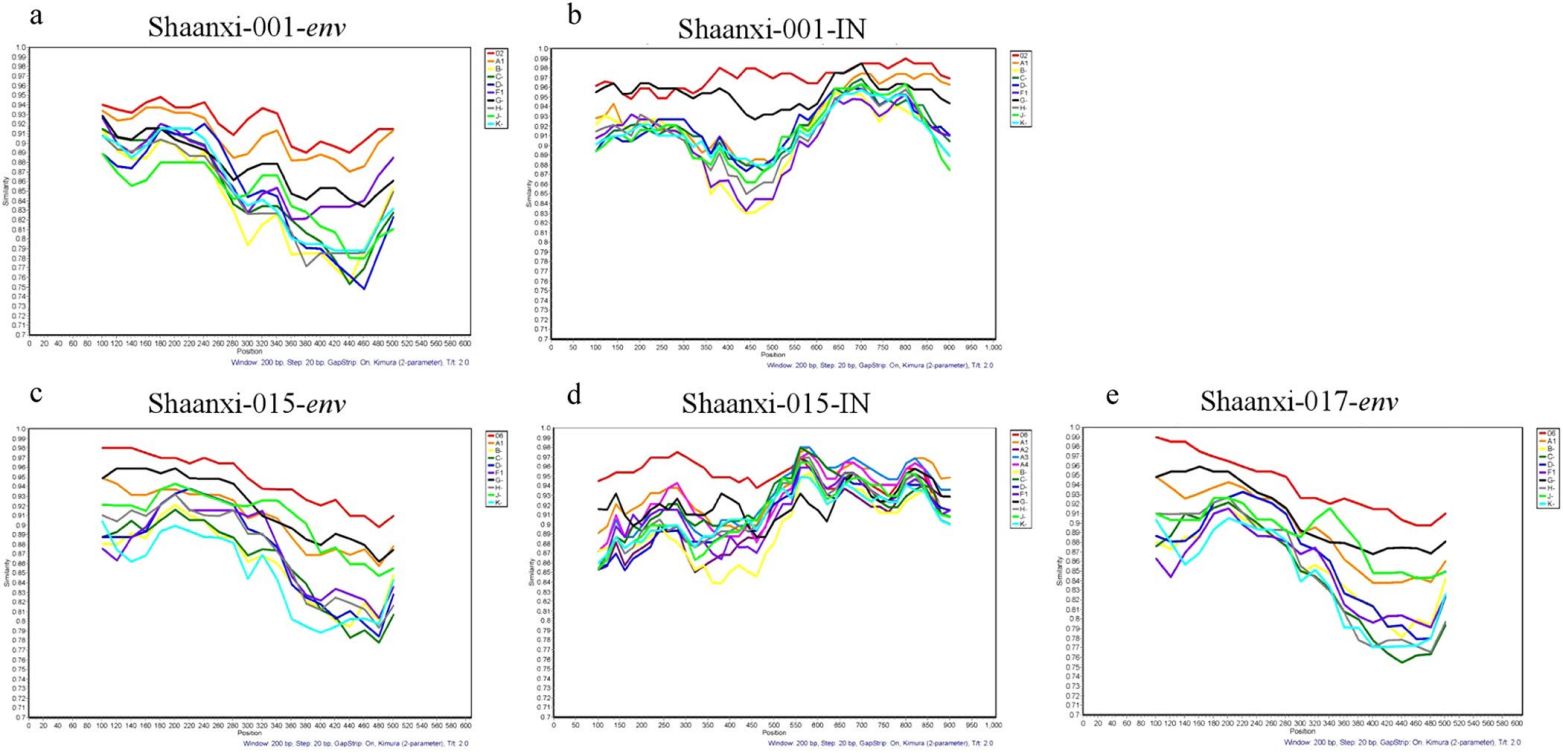
Similarity plots of the three rare recombinant partial-genome sequences from the HIV-1 infected blood donors. Each similarity plot was performed with Kimura-2 model of nucleotide substitution with a window size of 200 and a step size of 20. The color-coded key represents the different subtypes, sub-subtypes and CRFs of HIV-1. (**a**) Shaanxi-001 *env* sequence. (**b**) Shaanxi-001 IN sequence. (**c**) Shaanxi-015 *env* sequence. (**d**) Shaanxi-015 IN sequence. (**e**) Shaanxi-017 *env* sequence.

**Figure 5:**
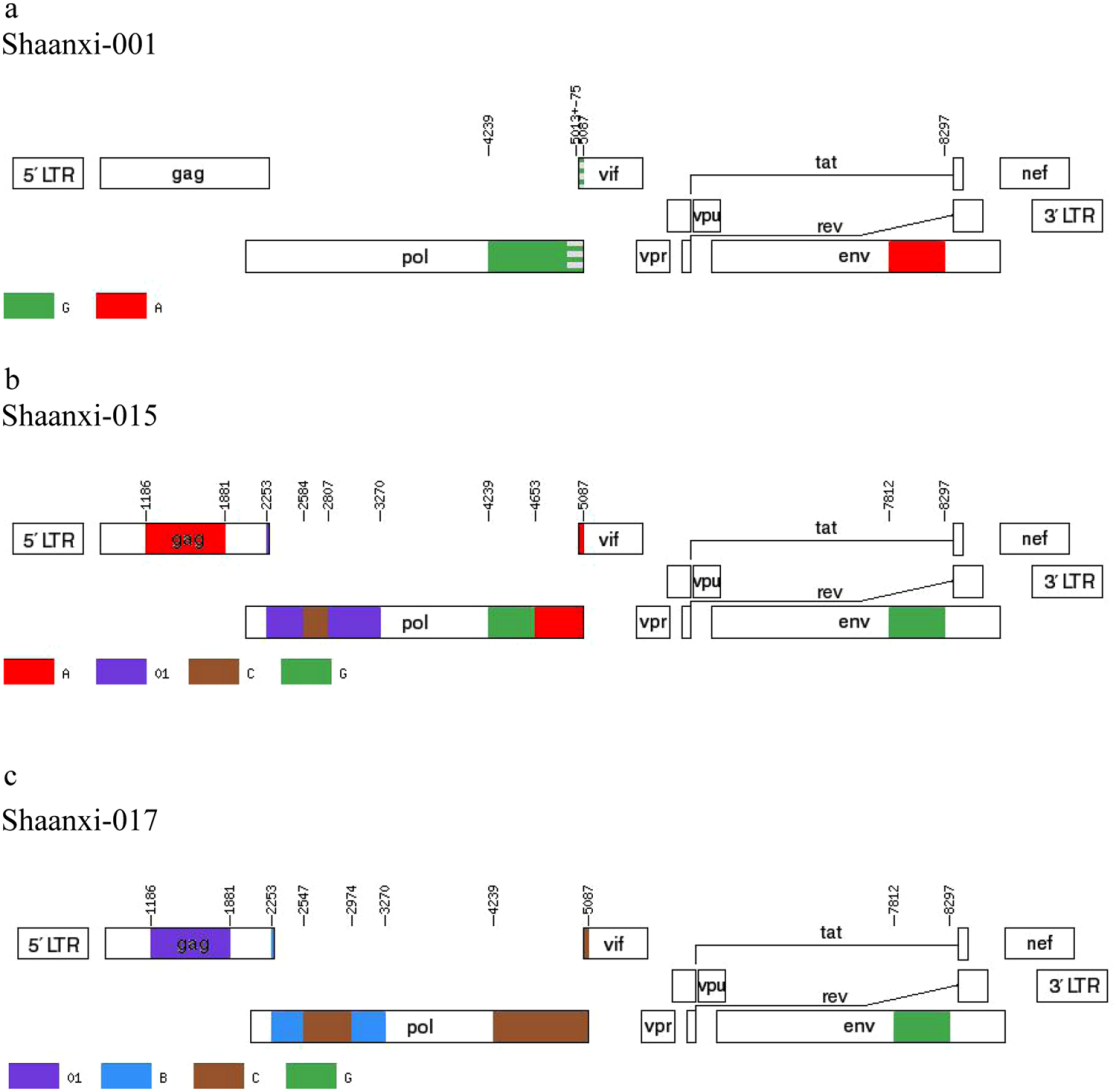
Three rare recombinant partial-genome maps. (**a**) Shaanxi-001. (**b**) Shaanxi-015. (**c**) Shaanxi-017.

Notably, several sequences (Shaanxi-001, Shaanxi-015, Shaanxi-017) were identified with rare classifications, CRF02_AG and CRF06_cpx, which are uncommon in China (Figs. 3 and 4). Of these, CRF06_cpx regions were present within two different URF strains, whereas the CRF02_AG sequences did not display evidence of recombination (Figs. 3 and 4). The three rare recombinant partial-genome maps were shown in Fig. 5. Furthermore, the details of bootscan and similarity analyses among the other 7 rare CRF strains including CRF55_01B, CRF59_01B, CRF65_cpx, CRF67_01B, CRF79_0107 and CRF85_BC subtypes were described in Figs. S2 and S3 respectively.

### ARV drug resistance–associated mutation analysis

The overall prevalence of DRMs was 15.6% (28/179) in this study population (Table 3). There were 4 (14.3%, 4/28) *protease* inhibitor (PI) accessory DRMs, 3 PI major DRMs and 22 (78.6%, 22/28) nonnucleoside *reverse transcriptase* inhibitors (NNRTI) DRMs. No accessory or major NRTI DRMs and *Integras*e Inhibitors (INSTIs) DRMs were found in these samples. The majority of blood donors with DRMs were infected with CRF07_BC or URF strains (60.7%, 17/28). Most of the PI accessory DRMs were Q58E (3 out of 4) and all the HIV-1 isolates with Q58E mutations in our study were CRF07_BC strains. The PI major DRMs included M46L, M46I and N88S. Most of the NNRTI DRMs (V179D/E [72.7%, 16/22]) were observed in HIV-1 infected blood donors and a combination of V179D and K103R were found in two samples may synergistically reduce ARV drug susceptibility. Furthermore, two blood donors with K103N mutation in the *reverse transcriptase* gene would be anticipated to have high-level resistance (HLR) to HIV-1 drug. Overall, the prevalence of primary DRMs among each geographic region was as follows, excluding the Northwestern District of China for small sample sizes (Table S1): North China: 14.8% (18/122), South China: 21.7% (5/23), Qinghai-Tibet region 16.1% (5/31).

**Table 3:** Characteristics of the blood donors identified with resistance-associated mutations.

Sample ID	Gender	Age	Donation	Ethnicity	Education	Genotype	PI accessory DRMs	PI major DRMs	NNRTI DRMs	Drug resistance
Henan-011	Male	24	First-time	Han	Associate degree	URF (PR-RT: CRF79_0107)	M46R			—
Henan-016	Male	46	Repeated	Han	Secondary school or below	CRF07_BC	Q58E			PLLR to NFV; LLR to TPV
Beijing-045	Male	34	Repeated	Han	Master/Bachelor degree	CRF07_BC	Q58E			PLLR to NFV; LLR to TPV
Shenzhen-010	Male	26	First-time	Han	Secondary school or below	CRF07_BC	Q58E			PLLR to NFV; LLR to TPV
Tongzhou-115	Male	29	First-time	Han	Associate degree	URF (PR-RT: B)		M46L	V106I	PLLR to ATV, FPV, IDV, LPV, SQV, TPV, ETR, NVP and RPV; LLR to NFV and DOR
**Harbin-007**	Male	19	First-time	Han	Master/Bachelor degree	CRF01_AE		M46I*		PLLR to ATV, FPV, IDV, LPV andSQV; IR to NFV
Shenzhen-011	Male	22	First-time	Han	Secondary school or below	CRF01_AE		N88S		HLR to ATV and NFV, LLR to IDV and SQV
**Tongzhou-017**	Male	31	First-time	Han	Associate degree	CRF07_BC			K101E*	IR to NVP and RPV; PLLR to DOR, EFV and ETR
**Tongzhou-025**	Male	53	First-time	Han	Associate degree	URF (PR-RT: CRF79_0107)			K103N*	HLR to EFV and NVP
**Tongzhou-126**	Male	39	First-time	Han	Associate degree	URF (PR-RT: CRF01_AE)			K103N^*^	HLR to EFV and NVP
Shenzhen-019	Male	31	First-time	Han	Master/Bachelor degree	CRF01_AE			V179D + K103R	IR to EFV and NVP; PLLR to ETR; LLR to RPV
Tongzhou-022	Male	43	First-time	Han	Associate degree	CRF65_cpx			V179D + K103R	IR to EFV and NVP; PLLR to ETR; LLR to RPV
Henan-005	Male	50	First-time	Han	Secondary school or below	URF (PR-RT: CRF07_BC)			V179D	PLLR to EFV, ETR, NVP and RPV
Henan-020	Male	35	First-time	Han	Secondary school or below	CRF07_BC			V179D	PLLR to EFV, ETR, NVP and RPV
Chongqing-008	Female	48	Repeated	Han	Secondary school or below	CRF07_BC			V179D	PLLR to EFV, ETR, NVP and RPV
Chongqing-015	Male	50	First-time	Han	Secondary school or below	CRF08_BC			V179D	PLLR to EFV, ETR, NVP and RPV
Changchun-066	Male	20	First-time	Han	Master/Bachelor degree	URF (PR-RT: CRF07_BC)			V179D	PLLR to EFV, ETR, NVP and RPV
Changchun-074	Male	24	First-time	Han	Associate degree	CRF01_AE			V179D	PLLR to EFV, ETR, NVP and RPV
Shaanxi-063-NAT	Male	28	First-time	Han	Secondary school or below	CRF07_BC			V179D	PLLR to EFV, ETR, NVP and RPV
Henan-019	Male	31	First-time	Han	Secondary school or below	URF (PR-RT: CRF55_01B)			V179E	PLLR to EFV, ETR, NVP and RPV
Chongqing-003	Male	57	Repeated	Han	Secondary school or below	CRF55_01B			V179E	PLLR to EFV, ETR, NVP and RPV
Chongqing-005	Male	26	First-time	Han	Secondary school or below	CRF08_BC			V179E	PLLR to EFV, ETR, NVP and RPV
Harbin-004	Male	29	Repeated	Han	Associate degree	B			V179E	PLLR to EFV, ETR, NVP and RPV
Harbin-022	Male	35	First-time	Han	Secondary school or below	URF (PR-RT: CRF55_01B)			V179E	PLLR to EFV, ETR, NVP and RPV
Shaanxi-022	Male	40	First-time	Han	Associate degree	CRF55_01B			V179E	PLLR to EFV, ETR, NVP and RPV
Shenzhen-012-2018	Male	29	First-time	Han	Secondary school or below	CRF55_01B			V179E	PLLR to EFV, ETR, NVP and RPV
Chongqing-011	Male	40	Repeated	Minority	Secondary school or below	CRF01_AE			V179T	—
Jiangsu-010	Male	39	First-time	Han	Associate degree	CRF01_AE			V179T	—

^*^Sequences with surveillance drug-resistance mutations (SDRMs).

1. The interpretation system reports five different possible levels of drug resistance(https://hivdb.stanford.edu/): Susceptible, Potential low-level resistance (PLLR), Low-level resistance (LLR), Intermediate resistance (IR) and High-level resistance (HLR).

2. Unique recombinant form (URF); *Protease* Inhibitors (PIs): Atazanavir (ATV), Darunavir (DRV), Fosamprenavir (FPV), Indinavir (IDV),Lopinavir (LPV), Nelfinavir (NFV), Saquinavir (SQV), Tipranavir (TPV); Non-nucleoside *Reverse Transcriptase Inh*ibitors (NNRTIs): Doravirine (DOR), Efavirenz (EFV), Etravirine (ETR), Nevirapine (NVP), Rilpivirine (RPV).

3. The combination of V179D and K103R defined as NNRTI DRM act synergistically to reduce EFV and NVP susceptibility.

4. M46R is a highly unusual mutation at this position, V179T is a relatively rare non-polymorphic mutation occasionally selected in patients receiving NNRTIs, the details about drug resistance are not shown in Stanford University HIV DRUG RESISTANCE DATABASE (https://hivdb.stanford.edu/).

TDR analysis in PR and PR by Calibrated Population Resistance tool showed that 4 HIV-1 isolates (2.4%, 4/168) had surveillance drug-resistance mutation (SDRM), including 3 NNRTI SDRMs (1 K101E, 2 K103N) and 1 PI SDRM (M46I) (Table 3). Of these 3 were collected from Beijing Tongzhou district blood bank, 1 was from Heilongjiang blood center.

## Discussion

During the past two decades, the HIV-1 epidemic has expanded from high risk groups (injection drug users, men who have sex with men, female sex workers *etc*.) to the general population, including blood donors^16^. Surveillance of the molecular epidemiology and diversity of HIV amongst blood donors is critical to determining the origin and evolution of HIV-1 variants in China and to prevention of transfusion-transmitted HIV-1 infections. Our study is the most geographically comprehensive epidemiological investigation of HIV among blood donors to date, encompassing 17 provinces and municipalities. The majority of the HIV-1 positive blood donors in the study were males with low educated level aged between 18–35 years. Prevention and screening strategies targeted towards these populations may have a greater impact towards ending the HIV pandemic.

Molecular characterization of the HIV-1 strains circulating within the blood donor population revealed unique patterns in comparison to other groups. Notably, subtype B and CRF08_BC accounted for 5.0% and 1.7% of blood donor infections, respectively, which was lower than the national prevalence reported in the 2006 survey^15^. Furthermore, the majority of subtype B strains (6/9) identified in this study were from Henan province, and 28.6% (6/21) of the HIV-1 strains from Henan blood centers were subtype B. During the mid-1990s, commercial plasma collection in central China (Henan and Shanxi provinces) led to an outbreak of subtype B among blood donors^17^. Since the prohibition of commercial blood collection, the prevalence of subtype B transmitted via blood transfusion has significantly decreased, which is reflected by the low proportion of subtype B among enrolled samples in this study. The absence of donor samples from Yunnan, Guizhou and Sichuan provinces in our study may have contributed to the observed low percentage of CRF08_BC, since CRF08_BC predominates in these provinces^17^. The geographic subtype distribution in Fig. 2 (excluding Qinghai-Tibet region: number of samples ≥10) indicated that CRF01_AE and CRF07_BC were the two main genotypes in each geographic region, which was consistent with previous studies amongst high-risk populations^15,18–23^. The presence of these same strains amongst the local blood donor population was evidence of the expansion of HIV-1 from high-risk groups to blood donor groups. This was consistent with the NHLBI Retrovirus Epidemiology Donor Study-II^9^, which also found many URFs in the Chinese blood donor population, indicating that ongoing HIV-1 recombination is becoming progressively complex in China. Due to the limited sampling of some blood centers and lack of samples from Qinghai-Tibet region in this study, we cannot make conclusions on the genotype distribution among blood donors throughout China. Future research must focus on expanded geographical coverage to get a more comprehensive dataset to improve blood safety, ART strategy and HIV control and prevention in China.

Our study was also consistent with previous work demonstrating that most Chinese URFs consist of CRF01_AE, B, and C regions (Table 2)^24,25^. However, the identification of two URFs containing CRF06_cpx regions and an ostensibly pure CRF02_AG infection are novel observations in a Chinese blood donor population. CRF02_AG is a subtype A/G recombinant form endemic to Africa^26^. Since the first identification of CRF02_AG in neighboring Taiwan in 1998^27^, several CRF02_AG variants have been reported in China^28,29^, but CRF02_AG has not previously been found amongst volunteer blood donors in China. Likewise, CRF06_cpx was first reported in Burkina Faso in 1998 and had circulated widely in West African countries^30^. Although a few CRF06_cpx isolates have been found in Beijing, Shenzhen and Hong Kong, this strain has not been reported in volunteer blood donors in China^31–33^. It was noteworthy that the CRF06_cpx regions found in Shaanxi blood donors in our study had recombined with CRF01_AE, A3, B and C strains. Therefore, it was likely that the recombination events that gave rise to these CRF06_cpx-containing URFs occurred in China. Complete genome sequencing of these strains and the CRF02_AG isolate will be required to identify all recombination breakpoints and estimate when these strains may have entered the Chinese blood donor population.

Generally, all HIV-1 positive blood donors are presumed to be treatment-naïve and the presence of ART resistant strains in this group is a reflection of the rate of transmitted drug resistance in a population^34^. DRM determinations were analyzed by Stanford HIVdb Program, which was based on subtype B and had biased the results for non-B strains^35^. In the present study, 15.6% (28/179) of HIV-1 infected blood donors had accessory or major DRMs. Notably, Q58E and V179D/E were the most common DRMs to PI and NNRTI respectively in the study, which is consistent with a previous study focused on five blood centers in China^9^. Q58E was identified as the potential low-level resistance (PLLR)-related muation to Nelfinavir (NFV) and low-level resistance (LLR)-related mutation to Tipranavir (TPV)^36^.

In particular, the Q58E DRM may be more common in CRF07_BC strains, which was also consistent with a recent study^9^. The PI major DRMs included M46L, M46I and N88S. M46I/L caused PLLR to many INSTIs among HIV-1 positive individuals, while N88S could result in HLR to Atazanavir (ATV) and NFV, LLR to Indinavir (IDV) and Saquinavir (SQV)^37–39^. Q58E and other DRMs (M46L/I, N88S) that confer resistance to PIs were present in our study. About PIs, only Lopinavir (LPV) was included in the Free AIDS Antiretroviral Therapy Manual, and not included in the first-line ART in China^40^, suggesting that either these DRMs were imported or they did not arise from selective pressure during treatment. In contrast, the most common NNRTI DRMs in our study, V179D/E mutations, were observed within a variety of strains, consistent with selective pressure from use of NNRTI in China^40,41^. A combination of RT V179D and K103R found in two samples with HIV-1 infection may synergistically reduce EFV and NVP susceptibility about 10-fold^42^, RT mutations with combination of V179D and K103R were also observed in treatment-naïve individuals in China^43^. The RT K103N mutation found in two strains can reduce EFV and NVP susceptibility by about 20- and 50-fold, respectively^44^.

Drug resistance analysis demonstrated that 2.4% of HIV-1 isolates contained at least one NNRTI (K101E, K103N) or PI (M46I) SDRMs, the overall prevalence of TDR was lower than previous reports in Zhejiang (11.1%) and Shijiazhuang (6.1%) among treatment-naïve HIV-infected individuals^45,46^, but similar to a nationwide cross-sectional survey about prevalence of TDR (3.6%) in 2015 in China^47^. Although the rate of TDR remained relatively low in Chinese blood donors in this study, the detection of 3 major NNRTI mutations and 1 PI mutation underlined the importance of a continuous surveillance of resistance mutations.

Overall, the prevalence of DRMs in South China was higher than other regions. The distribution of HIV-1 with DRMs in this study (Table S1) suggests that the HIV-1 strains isolated from positive blood donors in urban centers such as Beijing, Zhengzhou (Henan provincial capital) and Shenzhen had higher rates of DRMs. Moreover, 75% (3/4) HIV-1 isolates with SDRMs in the study were from Beijing Tongzhou district blood bank. It is possible that increased international travel and immigration in these populations may have contributed to the observed higher rates of DRMs.

In summary, our study characterized increasing HIV-1 diversity and high rates of drug resistance in the Chinese blood donor population, with unique province-level trends observed therein. The main HIV-1 subtypes of blood donors in most provinces were consistent with the local high-risk populations, suggesting that the HIV-1 epidemic has expanded from high risk groups to the general population. Most importantly, the integration of imported CRF02_AG and CRF06_cpx strains into the Chinese blood donor population is further evidence of the newly emerging migration patterns of the global HIV-1 pandemic. Furthermore, lots of DRMs and several TDR were found in treatment-naïve blood donors, underscoring the need for continued molecular surveillance to monitor and appropriately respond to expanding local HIV-1 diversity with diagnostic tests and therapeutics that are effective for circulating strains.

## Limitations

Since the prevalence of HIV-1 among Chinese blood donors remains low and not all the HIV-1 positive donations in blood screening laboratories were enrolled in our study, the limited sample sizes from several blood screening laboratories may bias the molecular epidemiological results. Furthermore, the socio-demographic data of the HIV-1 infected blood donor lack possible mode of transmission which would be used for analysis of the risks of HIV-1 transmission. For HIV-1 subtype analysis, HIV-1 genome sequencing including *env, pol and gag* genes is most reliable for subtype classification, but it’s hard to get HIV-1 genome sequence, due to the long length of genome sequence, low viral load in several samples.

Future research must focus on expanded geographical coverage and HIV-1 genome sequences to get a more comprehensive dataset to improve blood safety, ART strategy and HIV control and prevention in China.

## Materials and Methods

### Study samples

From January 2016 to December 2017, a total of 199 blood donations collected from 24 blood screening laboratories were confirmed as HIV viral load positive by the Abbott RealTime HIV-1 (Abbott Molecular Diagnostics, Des Plaines, IL, USA) test or serologically reactive by the Abbott ARCHITECT HIV Ag/Ab Combo test (Abbott Diagnostics, Weisbaden, Germany). These samples were tested non-reactive for Hepatitis B surface antigen (HBsAg), antibody to Hepatitis C Virus (anti-HCV) and antibody to *treponema pallidum* (anti-TP) in blood screening laboratories. Of these, at least two HIV regions were successfully sequenced for 179 plasma samples (Supplementary materials Table S1). Geographical localizationof blood screening laboratories in the study and the number of blood donations in these laboratories from January 2016 to December 2017 were shown in Fig. S4.

### RNA extraction, amplification and sequencing

HIV-1 RNA was extracted from 140 µL of HIV-1 positive plasma using QIAamp Viral RNA Mini Kit (Qiagen, Hilden, Germany) according to the manufacturer’s protocol. Amplifications of the HIV-1 *gag* p24, *env* gp41 and *polymerase* genes (*pol*) (encoding PR, RT and IN), were performed by QIAGEN OneStep RT-PCR Kit (Qiagen, Hilden, Germany) and nested PCR using AmpliTaq DNA Polymerase (Applied Biosystems, Foster City, USA). HIV-1 *gag* (HXB2: 1074–2047), PR-RT sequence (HXB2: 2068–3521), IN (HXB2: 4175–5214) and *env* gp41 (HXB2: 7648–8365) regions were amplified with outer primers respectively in the first round. The amplification of the *env* fragment was performed at 50 °C for 30 min for reverse transcription and then 95 °C for 15 min, followed by 50 cycles at 94 °C for 15 s, 50 °C for 30 s, and 72 °C for 1 min, with a final extension at 72 °C for 7 min. The other three regions were amplified following the same PCR conditions, except with an annealing temperature of 55 °C. The fist-round PCR products for four regions (5 µL) along with their respective inner primers were used in the nested PCR^48^. Nested PCR for *pol*-PR-RT was conducted with one cycle at 94 °C for 2 min, followed by 40 cycles at 94 °C for 30 s, 55 °C for 30 s, 72 °C for 1 min, and finally an extension of 10 min at 72 °C. Nested PCR for *gag*, IN and *env* genes followed the same procedure but with an annealing temperature of 50 °C. The nested PCR products were purified and sequenced by Sangon Biotech (Shanghai) Co., Ltd using Sanger methods. Details of the primers used in the study are described in Supplementary materials Table S2.

### HIV-1 genotype and phylogenetic analysis

The sample sequences were edited and aligned by Geneious 9.1.2 (https://www.geneious.com/products/prime/resources/download/previous-versions). *Gag* and two *pol* gene sequences covering PR and part of RT and the entire IN were submitted to the Los Alamos HIV BLAST tool for initial HIV-1 subtyping (https://www.hiv.lanl.gov/content/sequence/BASIC_BLAST/basic_blast.html)^49^ and then analyzed by REGA HIV-1 Subtyping Tool-Version 3.0 (http://dbpartners.stanford.edu:8080/RegaSubtyping/stanford-hiv/typingtool)^50^ and jpHMM program (http://jphmm.gobics.de/submission_hiv.html)^51^. Previous reports support utilizing the *env*, *gag* and *pol* regions for reliable subtype assignment^52–54^. The sequences were aligned with HIV-1 reference sequences (Accession numbers: Table S3) obtained from the Los Alamos database (https://www.hiv.lanl.gov) and then the nucleotide alignments were used to build phylogenetic tree for further HIV-1 subtyping by MEGA 7.0.2 (https://www.megasoftware.net) using the neighbor-joining algorithm based on Kimura 2-parameter model in 1000 bootstrap replicates^55^. The final subtype of HIV-1 isolate was confirmed by the consistent results from all the subtyping tools above. Boot-scanning and intra-genomic breakpoints analyses were conducted on sequences with possibly unidentified recombinant strains (the sequences with inconsistent results from all the subtyping tools above) through SimPlot 3.5.1 (https://www.softpedia.com/get/Science-CAD/SimPlot.shtml)^56^. Sequences with recombinant patterns that did not match established CRFs were classified as URFs^6,57^. The genome maps of URFs were generated through Recombinant HIV-1 Drawing Tool (https://www.hiv.lanl.gov/content/sequence/DRAW_CRF/recom_mapper.html). For finalization of phylogenetic classifications, neighbor-joining phylogenetic trees were prepared using PHYLIP 3.5 as previously described^58^ and simplified trees were visualized using FigTree v1.4.2 (University of Edinburgh, UK) to prepare figures.

### Drug resistance mutation analysis

DRM analyses in PR-RT and IN regions were performed in the Stanford HIV Drug Resistance Database (https://hivdb.stanford.edu)^59^ using Stanford HIVdb Program Genotypic Resistance Interpretation Algorithm (https://hivdb.stanford.edu/hivdb/by-sequences)^60^. Inferred levels of resistance of HIV-1 (PR, RT, IN) to 24 US Food and Drug Administration (FDA)-approved ARV drugs can be analyzed by the HIVdb program using total drug score derived by scores of each DRM related with the antiviral drug, and program reports are shown as the 5 levels of inferred drug resistance: HLR, intermediate resistance (IR), LLR,PLLR and susceptible^61^. Furthermore, populations of PR and RT sequences were submitted to CRP tool (http://cpr.stanford.edu/cpr.cgi) to perform standardized genotypic estimation of TDR^62^.

### Statistical analysis

Demographic data were obtained from the donor/donation database from each blood center and bank. SPSS 21.0 software was utilized for statistical analysis.

### Sequence data

The sequences described in this article have been deposited in the GenBank Nucleotide Sequence Database under accession numbers MK771158-MK771325 (*pol reverse transcriptase* sequences), MK771326-MK771493 (*gag* sequences), MK771495-MK771664 (*pol protease* sequences) and MK771494, MK771665-MK771829 (*env* sequences).

### Ethics approval and consent to participate

The study was approved by the Ethics Committee in Beijing Hospital (Ethics board approval number: 2016BJYYEC-118–01). Ethics statement in Chinese shown in supplementary material. The methods in the study were in accordance with the guidelines of the Declaration of Helsinki. Written informed consent was obtained from all subjects before blood donation.

## Supplementary information

Supplementary materials.

## Data Availability

The data for this study is available from the corresponding author on reasonable request.
