# Supplementary material for: HIV-1 molecular epidemiology and drug resistance-associated mutations among treatment-naïve blood donors in China
Source: Sci Rep. 2020 May 5;10:7571. doi: 10.1038/s41598-020-64463-w (PMC7200736; doi:10.1038/s41598-020-64463-w)
Supplement: Supplementary file 1 — Supplementary materials. [file 41598_2020_64463_MOESM1_ESM.pdf]

HIV-1 molecular epidemiology and drug resistance-associated mutations among treatment-naïve blood donors in China

Junpeng Zhao[1,2], Xiaoting Lv[3], Le Chang[1], Huimin Ji[1,2], Barbara J Harris[4], Lu Zhang[1,2], Xinyi Jiang[1,2], Fei Guo[1], John Hackett Jr[4], Peng Yin[4], Gavin A Cloherty[4], Mary A Rodgers[4], Lunan Wang[1,2*]

1 National Center for Clinical Laboratories, Beijing Hospital, National Center of Gerontology; Institute of Geriatric Medicine, Chinese Academy of Medical Sciences, P. R. China;

2 Graduate School, Peking Union Medical College, Chinese Academy of Medical Sciences, Beijing, P. R. China;

3 Abbott Laboratories, Research and Development, Shanghai, P.R. China;

4 Abbott Laboratories, Infectious Disease Research, Abbott Park, IL, USA.

[*] Correspondence: Lunan Wang PhD, National Center for Clinical Laboratories, Beijing Hospital, No.1 Dahua Road, Beijing 100730, P. R. China.

Mobile: 13910098136 Tel: 010-85133609

Postal address: No.1 Dahua Road, Beijing Hospital, Beijing, P. R. China.

Postal code: 100730

Email: lunan99@163.com

Supplementary materials

Table S1. The sources of HIV-1 infected blood donors

Geographic regions	Provinces or municipalities	HIV-1 positive	*gag* sequenced	PR-RT sequenced	IN sequenced	*env* sequenced	DRMs
North China	Beijing	13	13	13	13	13	6
	Heilongjiang	19	17	19	17	15	3
	Liaoning	11	11	9	10	11	0
	Shanxi	14	13	12	14	14	0
	Inner Mongolia	1	1	1	1	1	0
	Jilin	11	10	11	10	9	2
	Shaanxi	29	26	27	27	26	2
	Hebei	1	1	1	1	1	0
	Shandong	2	2	2	1	1	0
	Henan	21	20	20	21	21	5
South China	Hubei	2	1	1	2	2	0
	Jiangsu	10	10	9	9	9	1
	Guangdong	10	9	9	9	9	4
	Zhejiang	1	1	1	1	1	0
Northwestern District of China	Gansu	1	1	1	1	1	0
	Ningxia	2	2	2	2	2	0
Qinghai-Tibet region	Chongqing	31	30	30	31	30	5
Total		179	168	168	170	166	28

Table S2. PCR and sequencing primers.

Target region	Primers				
	Reaction	Direction	Name	Nucleotide sequence (5'-3')	Position (HXB2)
gag	RT-PCR	Forward	*gag*-F1	AGACACCAARGAAGCYTTAG	1074-1093
		Reverse	*gag*-R1	CTTCYTTYCCACATTTCCAAC	2047-2027
	Nested PCR	Forward	*gag*-F2	GCYTTAGABAARRTAGAGGAAG	1087-1107
		Reverse	*gag*-R2	GGCTAKGTGYCCTTCYTTG	1995-1976
	Sequencing	Forward	*gag*-F2	GCYTTAGABAARRTAGAGGAAG	1087-1107
		Reverse	*gag*-R2	GGCTAKGTGYCCTTCYTTG	1995-1976
		Forward	*gag*-F3	GTGAYATAGCAGGAACYACTAGT	1490-1512
PR-RT (Protease, Reverse transcriptase)	RT-PCR	Forward	RT-F1	ACTGARAGACAGGCYAATTTTTTAGGG	2068-2094
		Reverse	RT-R1	GTCTTTTGATGGGTCATAATAYRCTCC	3521-3495
	Nested PCR [48]	Forward	RT-F2	CAGAGCCAACAGCCCCACC	2147-2165
		Reverse	RT-R2	CTTCTGTATATCATTGACAGTCCAGCT	3326-3300
	Sequencing [48]	Forward	RT-F2	CAGAGCCAACAGCCCCACC	2147-2165
		Reverse	RT-R2	CTTCTGTATATCATTGACAGTCCAGCT	3326-3300
		Forward	RT-S-F1	GTTGACTCAGATTGGTTGCAC	2519-2539
		Forward	RT-S-F2	CCTAGTATAAACAATGAGACAC	2946-2967
		Reverse	RT-S-R1	GTGTCTCATTGTTTATACTAGG	2967-2946
IN (Integrase)	RT-PCR	Forward	IN-F1	TGGAGGRAATGAACAARTAGAT	4175-4196
		Reverse	IN-R1	TGGGATRTGTACTTCTGAACTT	5214-5193
	Nested PCR *	Forward	IN-F2	AACAAGTAGATAAATTAGTCAGT	4186-4208
		Reverse	IN-R2	ATACATATGRTGTTTTACTAARCT	5130-5107
	Sequencing *	Forward	IN-F2	AACAAGTAGATAAATTAGTCAGT	4186-4208
		Reverse	IN-R2	ATACATATGRTGTTTTACTAARCT	5130-5107
		Forward	IN-S-F1	TTAGCAGGAAGATGGCCAGT	4539-4558
env	RT-PCR *	Forward	*env*-F-1	TGARGGACAATTGGAGAARTGA	7648-7669
		Reverse	*env*-R-1	GGTGARTATCCCTKCCTAAC	8365-8346
	Nested PCR *	Forward	*env*-F-2	CAGCAGGWAGCACKATGGG	7798-7816
		Reverse	*env*-R-1	GGTGARTATCCCTKCCTAAC	8365-8346
	Sequencing *	Forward	*env*-F-2	CAGCAGGWAGCACKATGGG	7798-7816
		Reverse	*env*-R-1	GGTGARTATCCCTKCCTAAC	8365-8346

*: The primers were presented by ABBOTT Laboratories.

Table S3. Genbank Accession numbers for reference sequences.

Accession number	Classification	Accession number	Classification	Accession number	Classification
X52154	SIV	KC870030	CRF65_cpx	U63632	B
AF197340	CRF01_AE	KC183779	CRF67_01B	M17451	B
U51188	CRF01_AE	KC183780	CRF67_01B	AY173951	B
AF197341	CRF01_AE	KX673818	CRF77_cpx	U21135	B
U54771	CRF01_AE	KX673819	CRF77_cpx	U52953	C
AJ286133	CRF02_AG	KX673820	CRF77_cpx	AF067155	C
AF063224	CRF02_AG	KU161143	CRF78_cpx	AF110967	C
L39106	CRF02_AG	KU161144	CRF78_cpx	U46016	C
AF107770	CRF02_AG	KU161145	CRF78_cpx	AY772699	C
AJ288982	CRF06_cpx	KY216146	CRF79_0107	AY371157	D
AJ245481	CRF06_cpx	KY216147	CRF79_0107	U88822	D
AJ288981	CRF06_cpx	KY216148	CRF79_0107	U88824	D
AF064699	CRF06_cpx	KU820834	CRF83_cpx	K03454	D
AF286226	CRF07_BC	KU820842	CRF83_cpx	M27323	D
AF286230	CRF07_BC	KU820843	CRF83_cpx	AY253311	D
AX149647	CRF07_BC	KU820847	CRF83_cpx	AF005494	F1
AF286229	CRF08_BC	KU992928	CRF85_BC	AF075703	F1
AY008715	CRF08_BC	KU992929	CRF85_BC	AJ249238	F1
AY008716	CRF08_BC	KU992930	CRF85_BC	AF077336	F1
AY008717	CRF08_BC	AF484509	A1	AY371158	F2
AF516184	CRF15_01B	AB253429	A1	AF377956	F2
AF529572	CRF15_01B	AF004885	A1	AJ249236	F2
AF529573	CRF15_01B	AF069670	A1	AJ249237	F2
AF530576	CRF15_01B	M62320	A1	U88826	G
AY945734	CRF52_01B	AF286237	A2	AF084936	G
DQ354113	CRF52_01B	AF286238	A2	AF061641	G
DQ366664	CRF52_01B	AY521629	A3	AF061642	G
JX574661	CRF55_01B	AY521630	A3	AF005496	H
JX574662	CRF55_01B	AY521631	A3	AF190127	H
JX574663	CRF55_01B	AM000055	A4	AF190128	H
JX960635	CRF59_01B	AM000053	A4	AF082395	J
KC462190	CRF59_01B	AM000054	A4	AF082394	J
KC462191	CRF59_01B	AY331295	B	AJ249235	K
KC870027	CRF65_cpx	AY423387	B	AJ249239	K
KC870028	CRF65_cpx	K03455	B		

Fig S1

From: HIV-1 molecular epidemiology and drug resistance-associated mutations among treatment-naïve blood donors in China

a

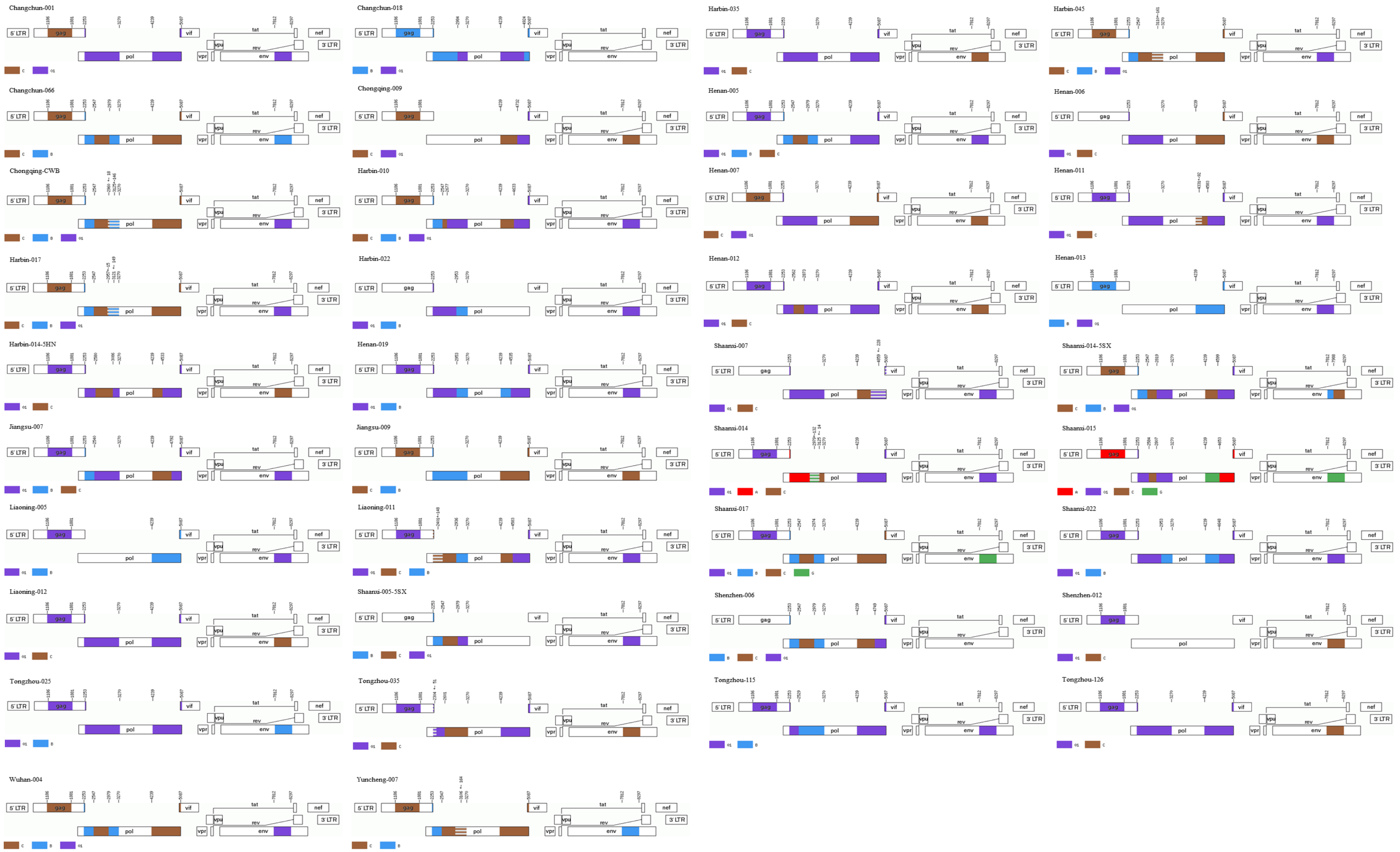

The genome maps of URFs. (a) 39 HIV-1 isolates were identified as URFs.

Fig S2

From: HIV-1 molecular epidemiology and drug resistance-associated mutations among treatment-naïve blood donors in China

a

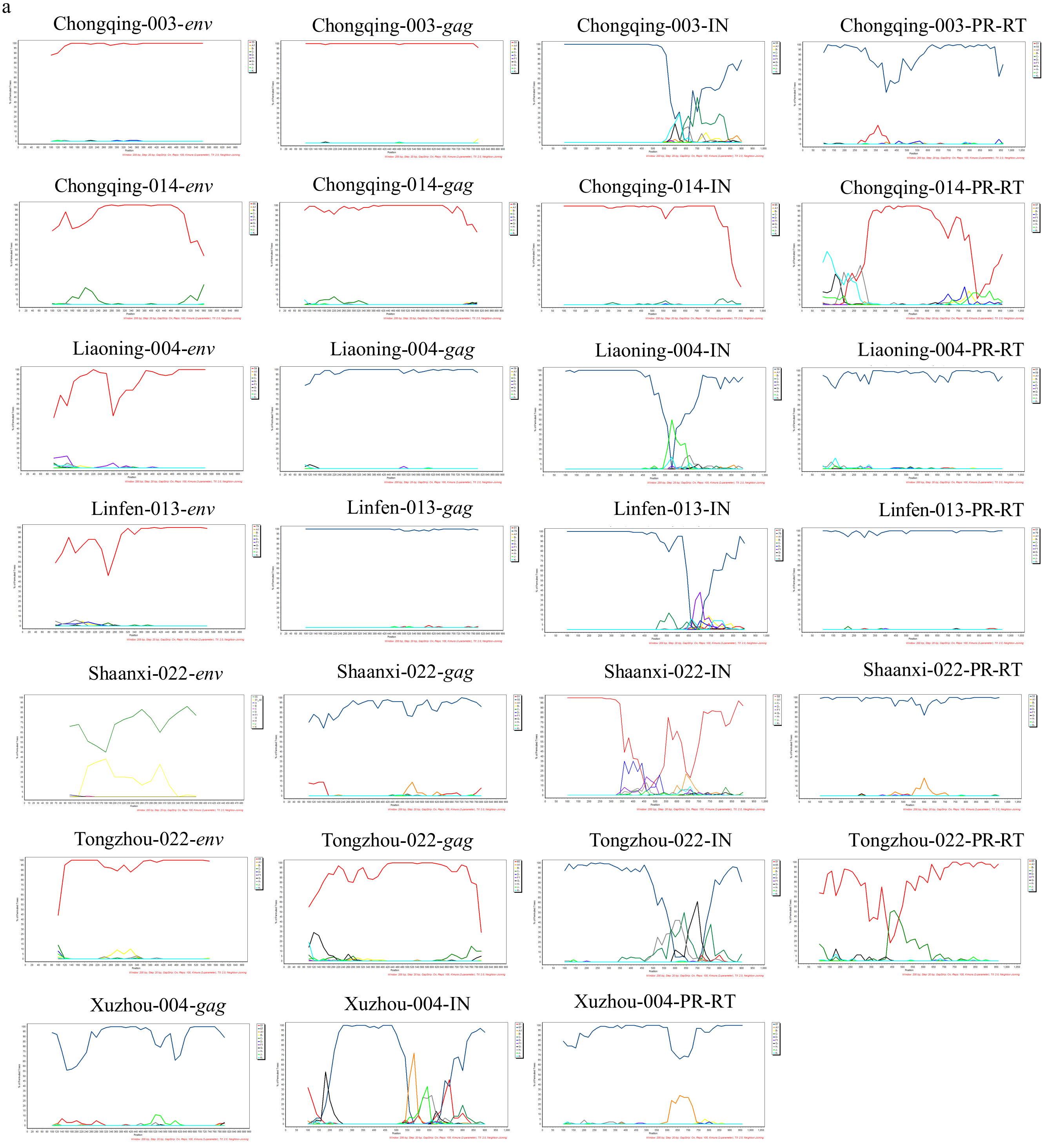

Bootscan plots of the 7 rare recombinant partial-genome sequences from the HIV-1 infected blood donors. (a) 7 HIV-1 isolates were identified as rare CRFs in China, *env, gag,* IN and PR-RT sequences among these strains were performed on bootscan analysis. Each bootscan plot was performed with Kimura-2 model of nucleotide substitution with a window size of 200 and a step size of 20. The color-coded key represents the different subtypes, sub-subtypes and CRFs of HIV-1. Subtype classification: Chongqing-003 (CRF55_01B), Chongqing-014 (CRF85_BC), Liaoning-004 (CRF59_01B), Linfen-013 (CRF79_0107), Shaanxi-022 (CRF55_01B), Tongzhou-022 (CRF65_cpx), Xuzhou-004 (CRF67_01B).

Fig S3

From: HIV-1 molecular epidemiology and drug resistance-associated mutations among treatment-naïve blood donors in China

a

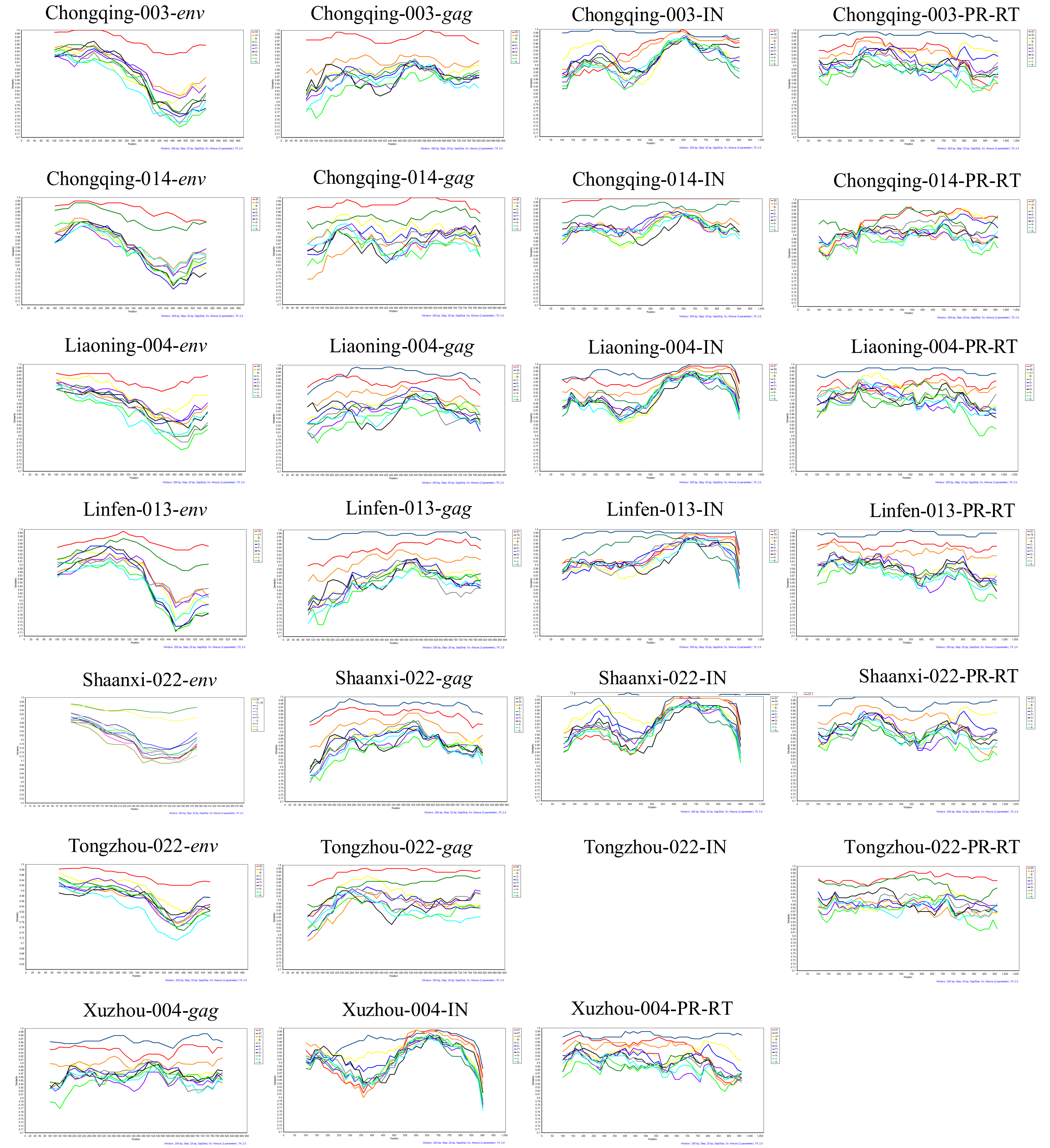

Similarity plots of the 7 rare recombinant partial-genome sequences from the HIV-1 infected blood donors. (a) 7 HIV-1 isolates were identified as rare CRFs in China, *env, gag,* IN and PR-RT sequences among these strains were performed on similarity analysis. Each similarity plot was performed with Kimura-2 model of nucleotide substitution with a window size of 200 and a step size of 20. The color-coded key represents the different subtypes, sub-subtypes and CRFs of HIV-1. Subtype classification: Chongqing-003 (CRF55_01B), Chongqing-014 (CRF85_BC), Liaoning-004 (CRF59_01B), Linfen-013 (CRF79_0107), Shaanxi-022 (CRF55_01B), Tongzhou-022 (CRF65_cpx), Xuzhou-004 (CRF67_01B).

Fig S4

From: HIV-1 molecular epidemiology and drug resistance-associated mutations among treatment-naïve blood donors in China

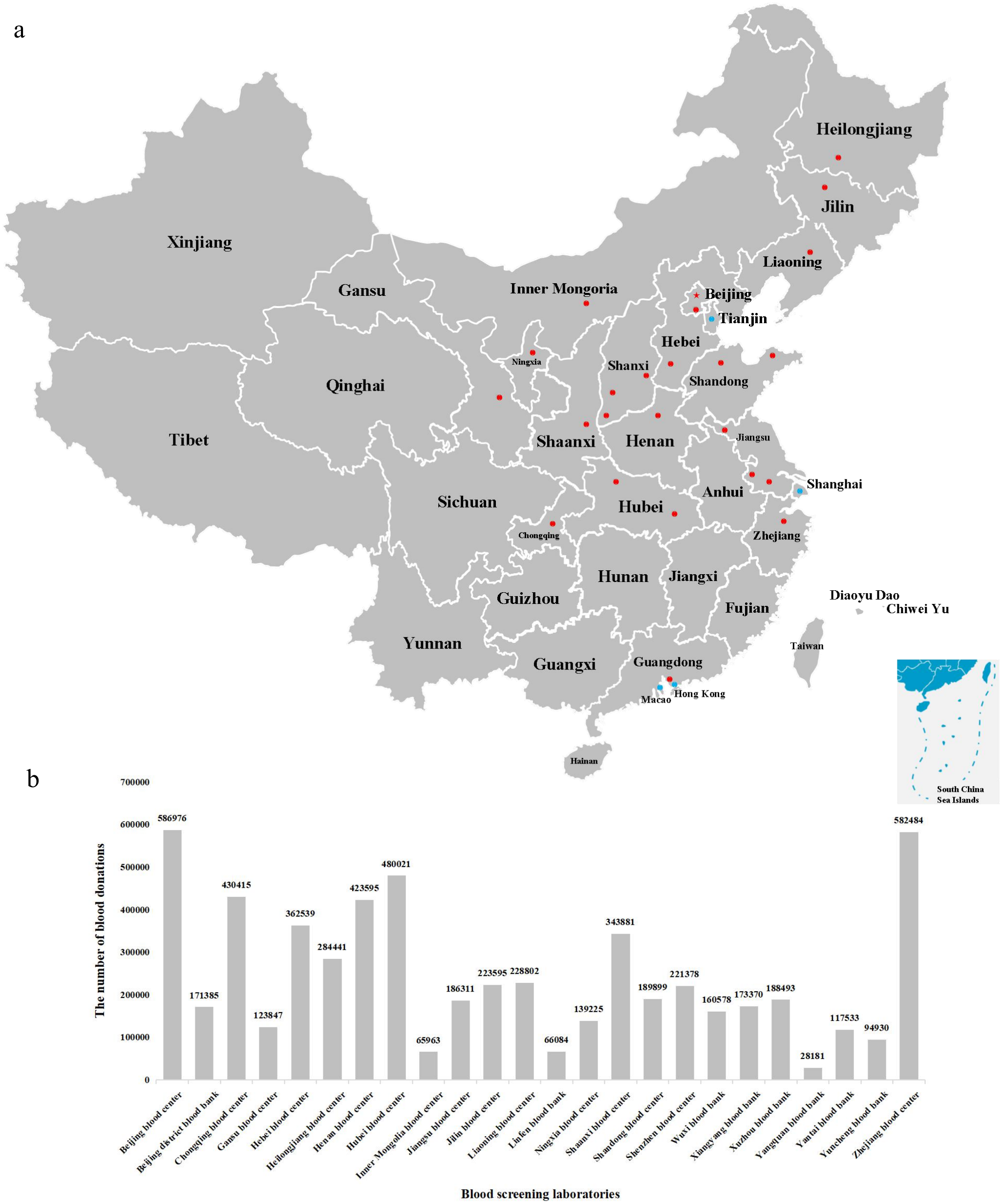

Geographical localization of blood screening laboratories in the study and the number of blood donations screened in these laboratories from January 2016 to December 2017. (a) 24 blood banks or blood centers were represented by red circles and pentagram. (b) The number of blood donations collected by the 24 blood screening laboratories.
